# Prostate Cancer Progression: as a Matter of Fats

**DOI:** 10.3389/fonc.2021.719865

**Published:** 2021-07-27

**Authors:** Natalia Scaglia, Yesica Romina Frontini-López, Giorgia Zadra

**Affiliations:** ^1^Biochemistry Research Institute of La Plata "Professor Doctor Rodolfo R. Brenner" (INIBIOLP), National University of La Plata/National Council of Scientific and Technical Research of Argentina, La Plata, Argentina; ^2^Institute of Molecular Genetics, National Research Council, Pavia, Italy

**Keywords:** lipid metabolism, fatty acids, prostate cancer, castration resistance, obesity, microenvironment, lipidomics

## Abstract

Advanced prostate cancer (PCa) represents the fifth cause of cancer death worldwide. Although survival has improved with second-generation androgen signaling and Parp inhibitors, the benefits are not long-lasting, and new therapeutic approaches are sorely needed. Lipids and their metabolism have recently reached the spotlight with accumulating evidence for their role as promoters of PCa development, progression, and metastasis. As a result, interest in targeting enzymes/transporters involved in lipid metabolism is rapidly growing. Moreover, the use of lipogenic signatures to predict prognosis and resistance to therapy has been recently explored with promising results. Despite the well-known association between obesity with PCa lethality, the underlying mechanistic role of diet/obesity-derived metabolites has only lately been unveiled. Furthermore, the role of lipids as energy source, building blocks, and signaling molecules in cancer cells has now been revisited and expanded in the context of the tumor microenvironment (TME), which is heavily influenced by the external environment and nutrient availability. Here, we describe how lipids, their enzymes, transporters, and modulators can promote PCa development and progression, and we emphasize the role of lipids in shaping TME. In a therapeutic perspective, we describe the ongoing efforts in targeting lipogenic hubs. Finally, we highlight studies supporting dietary modulation in the adjuvant setting with the purpose of achieving greater efficacy of the standard of care and of synthetic lethality. PCa progression is “a matter of fats”, and the more we understand about the role of lipids as key players in this process, the better we can develop approaches to counteract their tumor promoter activity while preserving their beneficial properties.

## Introduction

Prostate cancer (PCa) is the second leading cause of cancer death in men in the US and the fifth cause worldwide (1). While primary PCa is successfully treated with surgery, about 30% of PCa cases recur. Androgen deprivation therapy (ADT) is the standard of care for androgen-sensitive metastatic PCas (mASPC). mASPC are initially responsive to ADT but will eventually develop resistance, a disease stage known as metastatic castration-resistant PCa (mCRPC) (2). Management of mCRPC was primarily based on taxanes (i.e., Docetaxel, Cabazitaxel). However, in the last decade, second-generation androgen-receptor (AR) signaling inhibitors (i.e., enzalutamide) and intra-tumor androgen synthesis inhibitors (i.e., abiraterone) have been approved for the treatment of mCRPC with improved survival benefits. Unfortunately, efficacy is not long-lasting due to the occurrence of several mechanisms of resistance, including the overexpression of AR splicing constitutive active variants (i.e., AR-V7) (3). Therapeutic strategies based on the radioactive isotope radium-223 or cell-based immunotherapy (Sipuleucel-T) are also not resolutive (2, 4). Treatments with PARP inhibitors have been recently approved by the U.S. Food and Drug Administration (FDA) for the treatment of mCRPC patients harboring tumors with defects in DNA damage response (DDR), especially in the gene BRAC2, opening a new area for precision oncology in advanced PCa (5). This is also supported by the recent approval of two genetic tests, BRACAnalysis CDx and FoundationOne CDx to identifying mCRPC who have DDR genetic alterations and thus will most likely respond to PARP inhibitors (i.e., Olaparib and Rucaparib). (https://bit.ly/2z5Lu5C; https://bwnews.pr/2ZtfCSS). Clinical trials testing immune check-point inhibitors (ICI) are ongoing in mCRPC patients with disappointing results so far (6) Thus, strategies to boost responses to ICI are currently sought. Alterations of lipid metabolism in PCa cells were first observed long time ago using radiolabeling approaches and linked to AR signaling modulation (7, 8). However, the last decades have faced a change in the perspective of lipid role in cancer development and progression. In addition of being building blocks for membrane synthesis and energy fuel, lipids have emerged as key players in mediating oncogenic signaling, endoplasmic reticulum (ER) and oxidative stresses, non-apoptotic cell death (i.e., ferroptosis), and inflammatory stimuli (9). More recently, a lot of attention has been paid on the impact of lipids on the tumor microenvironment (TME), in particular on the immune TME (10). The plasticity of lipid metabolism rewiring allows PCa cells to thrive in hostile and nutrient-deprived environments and to spread to distant tissues. As a result, new mechanisms of therapy resistance and disease progression associated with lipid metabolism rewiring have recently emerged. This has also been supported by the recent advances in analytical techniques including high-resolution mass spectrometry (MS)-based lipidomics and MS-based imaging (MSI) that allow to measure hundreds of lipid species at once, Including rare lipid species, and to provide spatial resolution. These new technologies have uncovered the complexity and heterogeneity of lipid metabolism rewiring in a way that could not have been assessed before, opening new possibilities for both biomarkers and therapeutics discovery (9). Indeed, the combination of lipid metabolism modulators with standard of care is now strongly pursued.

In this review, we describe how lipid metabolism rewiring, including alterations in both *de novo* lipid synthesis, uptake, transport, storage, and utilization, contributes to PCa progression and therapy resistance and we discuss how these vulnerabilities can be exploited therapeutically. We emphasize the recently uncovered role of lipid metabolism in immune TME and the potential impact of *de novo* lipogenesis inhibitors as immunotherapy sensitizers. This review also briefly describes the advances in measuring and imaging lipids and how these more sophisticated analytical techniques contribute to improve biomarker discovery.

This review highlights that PCa progression is “a matter of fats” and lipids are rediscovered protagonists of oncogenic signaling, stress adaptation, and tumor-TME crosstalk. The more we understand about these aspects the better we can develop strategies to counteract their tumor supportive functions while enhancing their health-promoting roles.

## Lipid Metabolism Rewiring in PCa Development and Progression

Since the discovery in the mid-1990s of OA-519, an oncogenic antigen encoding for Fatty acid (FA) Synthase (FASN) highly expressed in breast cancer, research on the role of lipid metabolism in cancer has proceeded quite slowly with respect to other fields (11). However, interest for the mechanisms through which lipids promote tumorigenesis and tumor progression has been regained in the last decade, paralleled by the rapid development of high-resolution analytical techniques to interrogate the lipidome in a comprehensive and unbiased manner. While the majority of attention has been focused on the *de novo* FA synthesis dysregulation as hallmark of PCa development and progression, the perspective has lately changed to include many aspects of lipid metabolism spanning from FA uptake and transport, FA oxidation, lipid storage, and remodeling.

### Alterations in *De Novo* FA Synthesis

**Figure 1:**
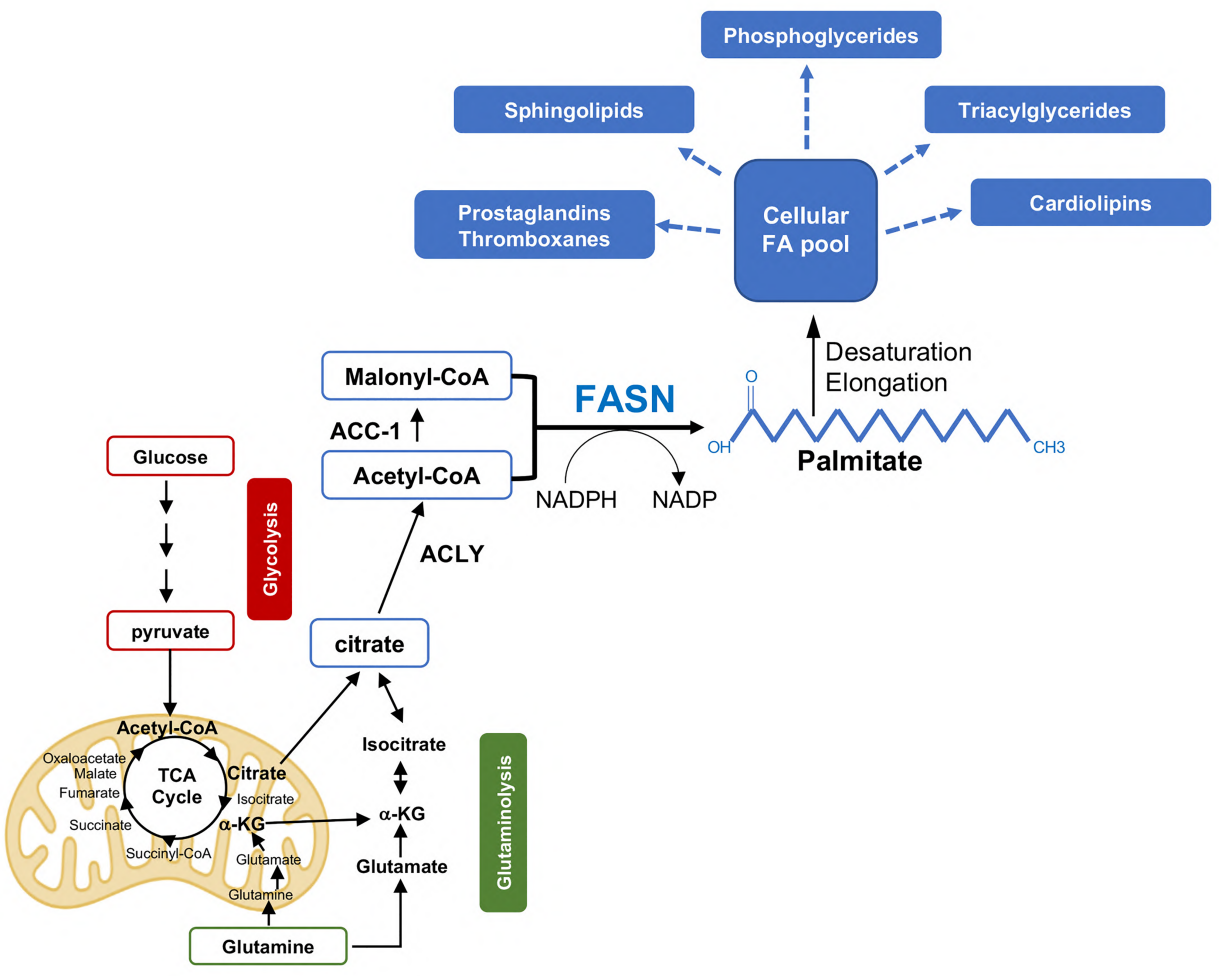
*De novo* FA synthesis in PCa progression. Acetyl-CoA derived from glucose and glutamine metabolism is exported to the cytosol in the form of citrate and reconverted to acetyl-CoA by the enzyme ATP citrate lyase (ACLY). Acetyl-CoA is then converted to Malonyl-CoA by the Acetyl coenzyme A carboxylase a (or ACC-1). Fatty acid synthase (FASN) catalyzes the condensation of Acetyl-CoA and Malonyl-CoA (in the presence of the reducing equivalent icotinamide adenine dinucleotide phosphate (NADPH)) to generate the 16-carbon FA palmitate, a saturated FA that undergoes further modifications (i.e., elongation/desaturation) to form more complex lipids. The latter serve as energy source, building blocks, and inflammatory/immune modulators to sustain PCa progression and CR. αKG, alpha-ketoglutarate; NADPH, Nicotinamide adenine dinucleotide phosphate.

While non-transformed prostate cells obtain the majority of lipids for membrane synthesis and energy fuel from the diet and circulation, PCa cells show an increased in *de novo* FA synthesis from glucose or glutamine, despite circulating lipids [reviewed in (12)]. This results in increased production of phospholipids and sphingolipids to support new membrane synthesis in proliferating PCa cells but also in a net accumulation of intra-tumor lipids mostly as triglycerides stored lipid droplets (LD) (13) (**Figure 1**). LD accumulation, which is associated with a more aggressive disease, provides an excellent reservoir for building blocks and energy in conditions of nutrients deprivation such as those encountered during PCa progression and metastatic spread. Moreover, LDs prevent lipotoxicity due to excessive accumulation of free FAs (see below). This increased net lipid production, known as “lipogenic phenotype”, is observed at early stages of PCa development and it is further enhanced in mCRPC. Consistently, enzymes or transcriptional factors (TFs) involved in *de novo* FA synthesis such as the TF sterol regulatory element-binding proteins (SREBPs), ATP citrate lyase (ACLY), Acetyl-CoA carboxylase (ACC), and FASN are overexpressed in primary PCa and especially in mCRPC. Specifically, FASN, the key lipogenic enzyme responsible for the synthesis of the 16C saturated FA palmitate from acetyl-CoA and malonyl-CoA, was found among the top ten genes overexpressed in AR-V7-driven CRPC metastases (mets) (14–16). In line with this, the interrogation of the Cancer Genome Atlas and other publicly available datasets uncovered a positive association between FASN expression and worse clinico-pathological features, including Gleason grade, tumor stage, lymph node positivity, shorter time to recurrence, cancer-free survival, and overall survival [reviewed in (17)]. As a result, great efforts are directed to exploit the lipogenic phenotype in mCRPC (see below). The work of Swinnen and coworkers has been instrumental to demonstrate the tight control of *de novo* FA synthesis by androgens/AR signaling, the major driver of PCa development and progression to mCRPC (7, 8). A feedforward mechanism between SREBP and AR was initially described, whereby AR promotes SREBP activation and nuclear translocation while SREBP regulates AR promoter activity and expression (18, 19). Later on, Chan and coworkers identified AR-binding site in FASN gene promoter, suggesting AR-mediated direct regulation of FASN expression (20). This evidence has been supported by immunoprecipitation sequencing (ChIP-seq) experiments that revealed AR binding sites in several lipogenic enzymes in CRPC samples, besides FASN (21). Altogether these data suggest that both indirect and direct mechanisms of AR-mediated control of the lipogenic program exist. Cai and coworkers analyzed AR cistrome and demonstrated that activation of lipid biosynthesis is a major function of AR signaling during PCa progression. Specifically, increased expression of AR-V7 turned out to be crucial for the reactivation of the lipid synthesis in CRPC, suggesting a key role of this splicing variant in regulating lipid metabolism in the CRPC setting. The authors also identified an AR-dependent lipogenic gene expression signature that predicts poor patient outcome (16). Our recent study has uncovered the existence of a reciprocal modulation between FASN and AR, in particular AR-V7, and it has proposed FASN inhibition as a non-canonical approach to indirectly antagonize AR-V7 and potentially overcome therapy resistance to enzalutamide and abiraterone (22). Overexpression of ACLY, ACC, and FASN has been consistently associated with increased PCa cell proliferation, tumor growth, migration and invasion, activation of oncogenic signaling, protection from chemotherapeutics-induced apoptosis, features that are reversed using genetic/pharmacological inhibition of enzyme activities [reviewed in (12)]. However, new roles for *de novo* FA synthesis in PCa progression have recently emerged. These involve post-translational modifications, DNA damage response, redox maintenance, ER and oxidative stress and resistance to ferroptosis, a lipid peroxidation-mediated non-apoptotic form of cell death [reviewed in (9)]. Palmitoylation of Wnt-1, RAS-related protein Rab-7a, alpha-tubulin, and eIF3L initiation factor are some of the post-translational modifications mediated by FASN and regulated by AR that activate oncogenic signaling in PCa (23–25). More recently, a palmitoyl-protein signature has been described in PCa derived extracellular vesicles (EVs), membrane-enclosed particles that play an important role in cancer progression as source of nutrients, signaling molecules, immune modulators, and circulating biomarkers, uncovering another potential mechanism of support to PCa progression (26).

In 2016, Wu and coworkers demonstrated the involvement of FASN in DNA repair and resistance to genotoxic insults. The authors found that FASN up-regulation regulates PARP-1 expression through NF-κB and SP1 modulation and increases Ku protein recruitment and DNA repair through activation of non-homologous end joining (27). Evidence for a direct interaction of FASN with MRN (MRE11-RAD50-NBS) complex has also been reported (28). By consuming NADPH, high rates of *de novo* FA synthesis also maintain redox balance and increased NADP/NADPH ratio, which is needed to support oxidative reactions such those in the pentose phosphate pathway for nucleotide synthesis (29). FASN expression/activity is also crucial in counteracting ER and oxidative stress in PCa by promoting saturated FA acids (SFA) synthesis and the remodeling of ER and mitochondrial membranes (22, 30–32). Furthermore, the increased production of SFAs and their acylation in phospholipids give rise to membranes characterized by a high ratio of SFA and polyunsaturated FAs (SFA/PUFA). These changes affect membrane fluidity, microdomains formation (i.e., lipid rafts), and lipid peroxidation (33, 34). SFA-enriched membranes affect the uptake of certain chemotherapeutics such as doxorubicin and promote the resistance to ionizing radiation (35, 36). Since SFAs are more resistant to lipid peroxidation, metastatic PCa cells with SFA-enriched membranes would be most likely less susceptible to oxidative stress-induced ferroptosis, an iron-dependent form of cell death induced by reactive oxygen species (ROS)-mediated lipid peroxidation (37). Targeting *de novo* lipogenesis and the Lands cycle has recently been shown to induce ferroptosis in KRAS-mutant lung cancer (38) and we anticipate similar results in mCRPC. These new findings have opened new possibilities for combinatorial treatments.

### Alterations in FA Modelling

**Figure 2:**
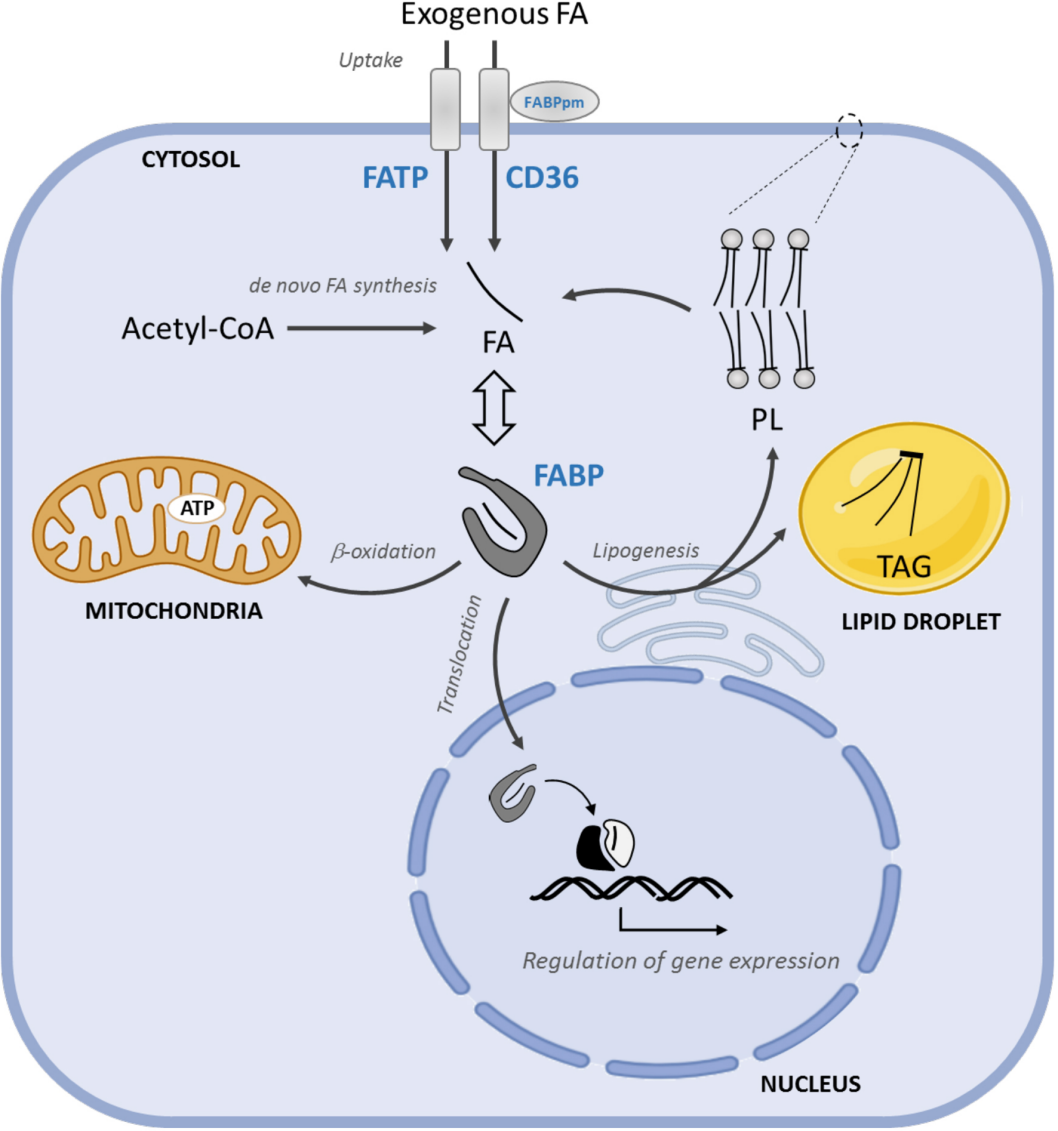
FA uptake, intracellular transport, and FAO in PCa progression. The uptake of exogenous FA is mediated by membrane FA transporters, CD36, FATPs and FABPpm. FABPs solubilize cytosolic FAs and coordinate their intracellular transport towards FABPs solubilize cytosolic FAs towards storage (mainly TAG) or structural (principally PL). Moreover, FABPs coordinate their intracellular transport lipids to the mitochondria for energy supply and to the nucleus where FA regulate gene expression storage (mainly TAG) or structural (principally PL) lipids, to the mitochondria for energy supply and to the nucleus, where FA regulate gene expression. FATP, FA transport proteins; FABPs, FA binding proteins; TAG, triacylglycerides; PL, phospholipids.

Once palmitate (16:0) and stearate (a 2C-elongated FA, 18:0), the most abundant SFAs, are synthesized or acquired from the diet (**Figures 1**, **2**) they usually undergo further modifications including desaturation and elongation. Desaturation of *de novo* synthesized SFAs involves the introduction of a cis-double bond to the acyl chain at the delta-9 (Δ9) position by stearoyl-CoA desaturases (SCDs) to generate the monounsaturated FAs (MUFA) palmitoleate and oleate (39). As humans lack delta-12 (Δ12) and delta-15 (Δ15) desaturases, PUFAs need to be acquired from the diet. Hence, α-linoleic acid (LA, an omega-6 PUFA) and α-linolenic acid (ALA, an omega-3 FA) are essential diet-derived PUFAs, which are required for the generation of further desaturated PUFAs (i.e., arachidonic acid), eicosanoids (i.e., prostaglandins and thromboxanes), and lipoxins, all of which play crucial roles as signaling molecules and mediators of PCa progression (40). LA and ALA desaturation is primarily catalyzed by the FA desaturases FADS1-3. Two human isoforms of SCD exist, SCD1 and SCD5 (41, 42). SCD1, the most abundant SCD in human cells, is highly expressed in human PCa with respect to normal tissues (43). Consistently, PCa cells upregulate *de novo* FA synthesis to generate SFA and MUFA-rich phospholipids that partition into detergent-resistant lipid rafts to markedly alter signal transduction, vesicular trafficking, and cell migration (44, 45). SCD1 pharmacological inhibition with BZ36 was shown to repress proliferation of AS LNCaP and CRPC C4-2 cells *in vitro* and *in vivo* through the abrogation of phosphatidylinositol generation and consequent inhibition of AKT pathway (46). Inhibition of SCD1 was also shown to activate 5' AMP-activated protein kinase (AMPK) and glycogen synthase kinase-3 (GSK3β), resulting in decreased β-catenin transcriptional activity (46).

SCD1 silencing also results in changes in the composition of cardiolipins, the major constituents of mitochondria membranes. As a result, alterations of mitochondria membrane favor the release of cytochrome c and the induction of apoptosis (43). Thus, overexpression of SCD-1 may represent a protective mechanism to apoptosis that PCa cells adopt, especially during stress. As an oxygen and NADPH-consuming process, desaturation occurrence is particularly challenging during cancer progression where hypoxic conditions are frequently observed. To overcome this, cancer cells tend to accumulate MUFAs in LDs, hydrolyze LDs, and assemble MUFA into PLs under hypoxic conditions. While the increase in MUFA incorporation in cellular membranes enhances their fluidity, it also reduces their PUFA/MUFA ratio, providing a robust protection from ferroptosis (47, 48). Thus, SCD-1 inhibitors are currently tested in the preclinical setting to induce ferroptosis (9, 48, 49).

Besides desaturation, FAs undergo elongation, a process that is catalyzed by a class of enzymes called elongases (ELOngation of Very Long fatty acids; ELOVLs), comprising seven members (ELOVL 1-7). ELOVLs add two carbon units to the carboxyl end of FA chains. While their precise functions are still not fully clarified, ELOVL-1, -3, and -6 predominantly elongate SFAs and MUFAs, ELOVL-2 and -4 elongate PUFAs, ELOVL-5 elongates MUFAs and PUFAs, and ELOVL-7 elongates SFAs and PUFAs (50). ELOVL-7 was the first elongase identified as overexpressed in human PCa tissues with respect to adjacent non-tumoral tissues (51). ELOVL-7 is induced by androgens and when overexpressed in LNCaP xenograft promotes tumor growth in mice fed high-fat diet (HFD). *In vitro* FA elongation assay and FA composition analysis showed that ELOVL-7 is preferentially involved in FA elongation of very-long-chain SFAs included in phospholipids and neutral lipids (i.e., cholesterol ester) and when silenced it reduces androgens synthesis and CRPC tumor growth (51). Both ELOV-7 and ELOVL-5 are among the lipogenic genes overexpressed following AR reactivation and thus considered critical for the progression to CRPC (16). Consistently, Centenera and coworkers showed a significant increase in AR-regulated elongation of fatty acyl chain phospholipids, mediated by ELOVL-5, in both PCa cells and patient-derived explants. ELOVL-5 silencing markedly altered mitochondrial morphology and function, leading to enhanced ROS generation and suppression of PCa cell proliferation, 3D growth, *in vivo* tumor growth, and metastasis formation. These features were rescued by the supplementation of cis-vaccenic MUFA, a direct product of ELOVL-5 elongation. These data suggest that lipid elongation is a metastasis-promoter metabolic pathway, which is targetable *via* ELOVL-5 (52). Aside from membrane lipid elongation, ELOVL-5 has more recently been involved in the generation of eicosanoids, inflammatory lipids with potent pro-tumorigenic signaling effects (9).

### Alterations in FA Uptake

The cellular uptake of free FAs, either derived from the hydrolysis of triacylglycerols (TAGs) in very low-density lipoproteins (VLDL), chylomicron or adipocytes, require their transport across the plasma membrane. The mechanisms and the identity of the proteins involved in this process are still not fully understood (53, 54). This is use, in part, to the use of bulky fluorescent, non-metabolizable FA analogs or indirect measurement of FA uptake. So far, the best characterized mediators of FA uptake are the scavenger receptor CD36, membrane-associated FA binding protein (FABPpm), and transmembrane FA transport proteins (FATPs) (**Figure 2**).

CD36 (also known as FAT, SCARB3, SR-B2, GP4 and others) is a ubiquitously expressed plasma membrane glycoprotein that binds diverse ligands, including FA, thrombospondin, oxidized low-density lipoproteins (LDL) and anionic phospholipids. CD36 is involved in FA uptake, clearance of apoptotic cells, and angiogenesis and it has been implicated in several diseases, including cancer (55–58). In skeletal muscles, CD36 has also been found in the outer mitochondrial membrane, where it might be involved in FA oxidation (FAO) under muscle contraction (i.e., exercise), although this aspect is still controversial (59, 60).

CD36 drives tumor progression in glioblastoma, melanoma, oral, and other carcinomas and it is required for stem cell self-renewal, tumor initiation, and metastatic potential in preclinical models (61, 62). CD36 is overexpressed more commonly in mets than in primary tumors and associated with poor prognosis (62–65). Furthermore, CD36 mRNA levels positively correlate with epithelial-mesenchymal transition (EMT) in several cancers, including PCa (63). In human PCa, CD36 protein was detected in both epithelial and stromal cells and equally expressed in tumor and adjacent normal regions, preventing its use as diagnostic biomarker (65). The discrepancy between the mRNA and protein findings may be ascribed to post-transcriptional mechanisms (66). Once in the cytosol, the fate of a FA largely depends on the cell metabolic status and ongoing signaling activation, resulting in FA incorporation in structural (mainly membrane PL) or storage lipids (in the form of TAG), in FA employment as second messenger or inflammatory molecule, and in FA use as fuel. The assessment of CD36 protein or mRNA levels has been used as FAO proxy in some studies. This *a priori* association is, however, often misleading, as in the case of PCa where enhanced FA uptake in human PCa and patient-derived xenografts (PDX) results in increased incorporation of FAs into complex lipids without FAO alteration. Consistently, CD36 ablation in PTEN knockout (KO) PCa mouse model failed to alter FAO (65). As expected, FA uptake impairment increases *de novo* FA synthesis as a compensatory mechanism, prompting the concomitant use of FA uptake and synthesis inhibitors. Accordingly, the combination of CD36 and FASN inhibitors significantly reduced PCa proliferation *in vivo* and in patient-derived PCa explants and it increased sensitivity to ionizing radiation, suggesting a potential synergistic effect in the clinical setting (65–68)

FABPpm is located in the outer plasma membrane leaflet and in mitochondria, displaying different function in each compartment (54). Despite the name, FABPpm is not related to the cytosolic FA binding proteins (see below). FABPpm expression is regulated by androgens in AS PCa cells, while its expression and function in CRPC cells is still largely unknown (69).

FATP1-6, also known as solute carrier family 27 (SLC27A1-6), are differentially expressed in a wide variety of tissues with different subcellular localizations. Their role as FA transporters and their function are still not fully clarified (54, 70). FATP1 is involved in FA metabolism and cancer progression (71–73). FATPs expression is highly heterogeneous in PCa tissues and cell lines and it varies across databases and detection methodologies (64, 74). The expression of FATP-6 is increased in enzalutamide-resistant LNCaP cells compared to the parental cells but no association with prognosis was observed in the clinical setting (75). Thus, further investigation on the role of FATP1-6 in PCa progression is required.

Besides those described above, other mechanisms for scavenging lipids from the extracellular *milieu* (76) may be involved. Recently, VLDL endocytosis has been described in breast cancer as a new mechanism to acquire exogenous FA, which may potentially occur also in advanced PCa (74, 77). The plasticity of cancer cells to obtain FA sources should be carefully taken into account during the planning and the design of therapeutic strategies. Dual targeting of FA uptake and synthesis holds promise for translation into the clinical setting.

### Alterations in FA Transport

Once in the cytosol, free FAs bind to FA binding proteins (FABPs), which increase their solubility in the intracellular aqueous *milieu*. FABPs are small (~15kDa) proteins that bind medium and long chain FAs as well as other lipophilic molecules, including eicosanoids, bile salts, lysophospholipids, and retinoic acid [reviewed in (78, 79)]. So far, ten different FABPs have been described in humans (FABP1-9, and the less characterized FABP12) showing tissue specificity and both redundant and distinctive functions (80). Acting as lipid chaperones, FABPs coordinate intracellular transport and lipid metabolism, and serve as sensors to signal FA supply to the nucleus (**Figure 2**).

FABP5 is the most characterized and highly expressed FABP in human PCas and cell lines, especially in CRPC cells (81–88). Different mechanisms account for FABP5 upregulation, including a positive feedback loop mediated by include proliferator-activated receptors (PPAR) PPARβ/δ (89), CpG island hypomethylation (85), and gene amplification (90). The latter is highly frequent in advanced CRPC (88). In human PCas, a positive correlation between FABP5 expression and androgen signaling responsive genes was observed. While FABP5 mRNA did not correlate with clinico-pathological features, FABP5 protein levels were significantly associated with high Gleason score and reduced patient survival (82, 84). Furthermore, FABP5 mRNA, protein, and serum levels were all increased in lymph node mets, suggesting FABP5 as a potential prognostic biomarker (91).

Consistent with a role in PCa progression, genetic or pharmacological inhibition of FABP5 decreased cell proliferation, colony-formation, invasive potential of PC3 and the more aggressive PC3-M cells. *In vivo*, tumor growth, mets formation, vascular endothelial growth factor (VEGF) expression, and microvessel density were also significantly reduced (81, 82, 92).

One of the main functions of FABPs is to escort both exogenous and *de novo* synthesized FAs towards nuclear receptors, such as the PPARs and modulate the expression of genes involved in cell survival, growth, migration, and invasion [reviewed in (93)]. A direct interaction between FABP5 and PPARβ/δ or PPAR*γ* has been demonstrated using *in vitro* and cell-based assays (94, 95) and it accounts for some of the pro-tumoral effects of FABP5 in PCa (89, 89, 95–98). In 2016, Forootan and coworkers showed that FABP5 promotes VEGF expression and angiogenesis through FABP5-mediated FA transport to PPAR*γ*. In CRPC, this mechanism overcomes the canonical AR-mediated regulation of VEGF/angiogenesis, suggesting FABP5/FA/PPAR*γ* pathway as a potential therapeutic target (97). PPAR-independent FABP5-mediated regulation of gene expression has also been described in PCa (98, 99). Other FABP5-mediated oncogenic mechanisms include the activation of SREBP-1c and the hypoxia-inducible factor 1-alpha (HIF-1α), although their roles in PCa has not been explored yet (100, 101).

FABP5 is also secreted by adipocytes, and it may potentialy contribute to the tumor supportive role of periprostatic fat (102). Finally, FABP5 has been found in urinary extracellular vesicles, where it may serve as a prognostic PCa biomarker (103).

Both pro-tumorigenic and anti-tumorigenic roles have been ascribed to FABP4 according to tumor type and TME. While FABP4 acts as a tumor suppressor when ectopically expressed in DU145 PCa cells, recent studies suggest FABP4 involvement in adipose-PCa crosstalk. According to this model, FABP4 promotes FA release from adipocyte TAG to fuel mets formation while cancer cells induce changes in adipocyte metabolism to promote FA release (104, 105). Consistently, Herroon and collaborators showed that adipocyte-derived conditioned media increases FABP4 and CD36 expression in PCa and breast cancer cells, their proliferation, invasion, and LD accumulation. This was also associated with a significant increase in several cytokines, VEGF, and HIF-1α. Conversely, inhibition of FABP4 impaired adipocyte-derived conditioned media-induced invasion (106). Furthermore, HFD was shown to induce FABP4 expression in PC3 bone tumors but not in subcutaneous ones, indicating that bone marrow-derived adipocytes may promote specific metabolic alterations in PCa bone mets (106). Oncomine data and immunohistochemistry (IHC) confirmed increased expression of FABP4 mRNA and protein in bone mets, especially in areas enriched for infiltrating adipocytes (64, 106). Mechanistically, FABP4 expression is dependent on PPARγ, which in turn is activated by FA/FABP4, suggesting the existence of a feedforward mechanism that sustains high FABP4 levels in PCa cells.

Similar to FABP5, FABP4 is also secreted by adipocytes and it plays a role as adipokine. Circulating FABP4 levels correlates with obesity and some features of the metabolic syndrome in both mice and humans (107–109) and may impact PCa progression. Indeed, serum FABP4 levels were associated with high Gleason grade (110, 111). Several evidence suggest a link between FABP4 intracellular levels and PCa aggressiveness. Ectopic expression of FABP4 promotes DU145 PCa cell invasion *in vitro*, while *in vivo* FABP4 knockdown (KD) reduces tumor growth and lung mets formation (112). Haung and coworkers also uncovered FABP4-mediated tumor/TME crosstalk that sustains PCa invasive potential. According to this, not only PCa-secreted FABP4 increases PCa invasiveness by upregulating matrix metalloproteinases (MMP 2 and 9) but it also induces stromal cells to secrete interleukin-8 and -6, further promoting PCa invasiveness (110). Conversely, FABP4 inhibition was shown to decrease HFD-induced mets, adipocyte infiltration, reactive fibroblasts and serum IL-8. Altogether, these data support a critical role for FABP4 in shaping the TME and promoting PCa progression (110). Since both FABP 4 and 5 are also expressed in macrophages and endothelial cells, they may contribute to tumor-TME crosstalk through other mechanisms.

Although less characterized, other FABPs are involved in PCa onset and progression. FABP4, FABP5, FABP8, FABP9, and FABP12 loci were found in a commonly amplified region within the chromosome 8 (8q21.13), frequently observed in human PCas mets. In line with this, increased mRNA levels of FABP 4, 8, 9, and 12 were associated with increased Gleason score and PCa recurrence (90). In 2020, Liu and coworkers also demonstrated that FABP12 promotes EMT and PCa cell motility, at least in part, through a PPAR*γ*-dependent pathway (113), while FABP9 suppression inhibits PC3 cell invasive potential in PPAR*γ*-independent manner (86).

Altogether these data strongly support a role for FABPs in PCa progression and their potential use as therapeutic targets (9).

### Alterations in FA oxidation

While the majority of reports describe AR-mediated regulation of *de novo* FA synthesis in PCa progression, evidence is accumulating that both FA synthesis and FAO are regulated by AR signaling and contribute to castration resistance (CR) in a fine-tuned manner. For FAO to occur, FAs need to be converted to fatty acyl-CoAs by long chain Acyl-CoA Synthetases (ACSLs) and to cross the outer mitochondrial membrane. The latter is mediated by Carnitine palmitoyltransferase 1 (CPT-1), specifically the isoform CPT-1A. CPT-1A allows FA-CoAs across the mitochondrial membrane through the conversion to FA-carnitine, a rate-limiting step for FAO [reviewed in (12, 114)]. FAO is transcriptionally regulated by the PPAR family which mainly activate the expression of CPT-1 and other FAO enzymes in response to glucose deficiency, and post-translationally *via* the allosteric inhibition of CPT-1 by malonyl-CoA. The latter is mediated by the activation of AMPK, which phosphorylates and prevent ACC-2 (or ACCβ, the isoform expressed in the mitochondria) to synthesize malonyl-CoA [reviewed in (12, 114)]. Once in the mitochondria, FAs are oxidized to acetyl-CoA, which is used for energy production, generation of reducing equivalents to maintain redox homeostasis, or as substrate for new anabolic processes. During hypoxia or in response to drug treatment, cancer cells appear to favor FAO to rapidly generate ATP and NADH and promote survival. Indeed, targeting FAO with etomoxir was shown to reduce hypoxic areas in combination with radiation in metastatic PCa sphere (115).

The group led by Schlaepfer has been instrumental in uncovering the role of FAO in PCa progression to CRPC. In 2017, the authors showed that CPT1A isoform is abundant in high-grade PCa compared to benign tissues, and they demonstrated a synergistic effect in combining CPT-1A inhibitors with anti-androgen therapy. Mechanistically, the authors uncovered that CPT1A inhibition decreases AKT and inositol polyphosphate-5-phosphatase K (INPP5K) activation, resulting in increased AR activity and sensitivity to enzalutamide. Combination of FAO inhibitors (etomoxir, ranolazine, and perhexiline) with enzalutamide displayed a synergistic inhibitory effect, suggesting that co-targeting FAO and AR may have anti-cancer efficacy in mCRPC clinical setting (116). In 2019, the same group provided evidence for a new link between FAO and CR. The authors demonstrated that androgen withdrawal (which mimics the standard of care therapy for metastatic PCa) increases CPT-1A expression and FAO activity, which supports CRPC growth and antiandrogen resistance by supplying acetyl groups for histone acetylation (117). In a follow-up study, the authors showed that CPT-1A overexpression promotes antioxidant defenses, which foster PCa progression (118). Finally, last year, the group put forward the involvement of FAO in immunomodulation. Using the TRAMPC1 PCa model, the authors demonstrated that FAO inhibition with ranolazine decreases Tim3 content in CD8+ tumor-infiltrating T cells, increases macrophages, and decreases blood myeloid immunosuppressive monocytes, suggesting that targeting FAO stimulates anti-cancer immunity (118).

Besides CPT-1A, other FAO enzymes are involved in PCa progression. Combining proteomics and metabolomics, Biomme and coworkers identified the mitochondrial 2,4-dienoyl-CoA reductase (DECR1), an auxiliary FAO enzyme, as critical for CRPC. DECR1 participates in redox homeostasis by controlling the ratio between saturated and unsaturated phospholipids. As a result, DECR1 KO induced ER stress and sensitized CRPC cells to ferroptosis. Furthermore, DECR1 deletion impaired lipid metabolism and reduced CRPC tumor growth *in vivo* (119). Similar results were obtained by Nassar and coworkers using different models. The authors confirmed DECR1 KD-mediated cellular accumulation of PUFAs, enhanced mitochondrial oxidative stress, and lipid peroxidation. Specifically, DECR1 KD selectively inhibited PUFA oxidation, resulting in the suppression of proliferation, migration of PCa cells (including those resistant to enzalutamide), and metastasis formation in mouse xenograft models (120). These new findings implicate PUFA oxidation *via* DECR1 as an unexplored facet of FAO to promote PCa progression.

Yajun and coworkers also uncovered the involvement of the FAO regulator nuclear envelope protein Sun2 in PCa progression. The authors found a reduction of Sun2 expression in PCa tissues compared with adjacent normal tissues, which correlated with higher Gleason grade, postoperative T stage, lymph node invasion, and shorter PCa-free and overall survival. Sun2 silencing increased FAO activity, feature that was reversed by the use of etomoxir, suggesting a new role for Sun2 in promoting PCa progression through FAO modulation (121). Finally, Itkonen and coworkers identified enoyl-CoA-isomerase 2 (ECl2), a novel AR target involved in FAO. ECl2 was found overexpressed in PCa samples and associated with poor outcome, suggesting its possible involvement in PCa progression (122).

### Alterations in Lipid Storage

Under excess of nutrients, *de novo* or acquired FAs are incorporated in TAGs and accumulate as LDs, organelles composed by deposits of TAGs and cholesterol esters, and surrounded by a monolayer of PLs. LDs represent a reservoir and source of lipids for cancer cells, particularly under stress conditions such as hypoxia (123). Increased abundance of LDs is a feature of many aggressive cancers, including PCa [reviewed in (9)]. The terminal step in TAG biosynthesis is catalyzed by acyl-CoA:diacylglycerol acyltransferase (DGAT) enzymes, which transfer an acyl chain from fatty acyl CoA to diacyl glycerol (DAG). DGAT1 is overexpressed in PCa compared to normal epithelium and a recent study demonstrated that inhibition of DGAT1 reduces cell proliferation and migration *in vitro* and tumor growth *in vivo* by regulating intracellular lipids and non-centrosomal microtubule-organizing center (MTOC) protein GM130 (124). Similar results were also independently obtained by Mitra and coworkers (125). Using label-free Raman spectroscopy, Yue and coworkers demonstrated an aberrant accumulation of esterified cholesterol in LDs in high-grade PCa and mets due to the loss of the tumor suppressor PTEN, the activation of PI3K/AKT pathway, and the consequent activation of TF SREBP and LDL receptor (LDL-R). LD accumulation required the occurrence of cholesterol esterification. As a result, pharmacological and genetic inhibition of cholesterol esterification using cholesterol acyltransferase (ACAT) significantly suppressed cancer proliferation, migration, invasion, and tumor growth *in vivo* (126). This finding suggests ACAT as a potential target in PTEN mutated/deleted CRPC, which account for around 70% of CRPCs.

TAGs in LDs are sequentially hydrolyzed by three different lipases, the adipose triglyceride lipase (ATGL), the HS lipase (HSL), and the monoacylglycerol lipase (MAGL) [reviewed in (9)]. In 2011, Nomura and coworkers showed that MAGL is increased in androgen-independent human PCa cell lines, and that pharmacological or genetic inhibition of MAGL impairs PCa aggressiveness. Furthermore, MAGL was found as part of an EMT and stem-like gene signature, suggesting MAGL as a potential therapeutic target in advanced PCa (127). These data highlight LDs are critical players in supporting PCa progression, especially under stress.

### Alterations in Phospholipid Synthesis and Membrane Remodeling

FAs are essential building blocks for PLs. Early studies showed that a substantial fraction of the FAs acquired by PCa end up in PLs, which together with cholesterol and sphingolipids are the major constituents of membranes. In 2003, Swinnen and coworkers demonstrated that FASN plays a major role in the synthesis of PLs partitioning into detergent-resistant membrane microdomains, the latter being involved in key cellular processes including signal transduction, intracellular trafficking, cell polarization, and cell migration (45). PLs can be synthesized *de novo* but they can also be dynamically remodeled. For *de novo* PL synthesis, FAs are first incorporated in phosphatidic acid (PA) followed by phosphatidylcholine (PC), and phosphatidylethanolamines (PE) synthesis through the Kennedy pathway, although PE can also be generated from phosphatidylserines (PS) by headgroup exchange. PS is synthesized in the ER by headgroup exchange from PC and PE. Phosphatidylinositol (PI) is indirectly synthesized from PA, while cardiolipins (CL) are synthesized locally [reviewed in (9)]. PLs remodeling is catalyzed by phospholipases which can release acyl chains at different positions depending on the subclass of enzymes (PLA, PLC, PLD), while PL reacylation is catalyzed by a class of acyltransferases such as lysophosphatidylcholine acyl transferases (LPCAT). Our group demonstrated that *de novo* PC synthesis is required for cell cycle completion, upon cell division (128). Many enzymes involved in PL synthesis and remodeling are highly dysregulated in PCa. Lipin-1, a phosphatidic acid phosphatase (PAP) that regulates the rate-limiting step in PL synthesis is overexpressed in high-grade PCa and in PCa cells resistant to chemotherapy (i.e., Docetaxel). cBioPortal data also showed that patients with Lipin-1 amplification are characterized by decreased survival. Lipin-1 KD decreased both PCa cell proliferation and migration through RhoA activation, increased PA levels, and induced autophagy through the inhibition of PI3K/AKT/mTORC1 pathway., Lipin-1 depletion with propranolol sensitized cancer cells to rapamycin, suggesting new combination therapies (129, 130). Choline kinase alpha (ChoKa), the first enzyme of the Kennedy pathway, is also overexpressed in several cancer including PCa (131). Priolo and coworkers showed that the oncogene MYC increases ChoKa expression, as well as lipid synthesis (132). In line with this, positron emission tomography (PET) with PL-precursors ^11^C-choline or ^18^F-fluoro-choline has shown promising results in the detection of PCa recurrence and mets (133). Asim and coworkers demonstrated that ChoKa expression is regulated by androgens and it is positively associated with tumor stage. The authors also uncovered a role for ChoKa as a chaperone that binds to AR ligand-binding domain (LBD), enhancing AR stability. Consistently, ChoKa inhibition decreased AR protein levels and AR transcriptional program, and inhibited the growth of PCa cell lines, human PCa explants, and tumor xenografts (134), suggesting ChoKa as a marker of tumor progression and a potential therapeutic target. PLs remodeling is catalyzed by phospholipases, including PLA2, which is also involved in the generation of signaling FAs such as arachidonic acid (AA, see below) and lysophospholipids (LysoPLs). Phospholipase A2 Group IIA (PLA2G2A), especially the secretory form, is overexpressed in almost all human PCa specimens and correlate with high tumor grade. Blocking sPLA2-IIa function compromises CRPC cell growth, highlighting sPLA2 as a potential therapeutic target for CRPC. Serum sPLA2-IIa levels were increased in PCa patients and associated with high Gleason score and advanced disease stage, suggesting that serum sPLA2-IIa may serve as a PCa prognostic biomarker. A recent report also associated the expression of PLA2G2A with ferroptosis resistance through PUFA depletion in PCa membranes (135, 136). LysoPLs can stimulate PCa cell migration through several mechanisms, including the activation of the cationic channel T transient receptor potential vanilloid 2 (TRPV2), and the activation of lysophosphatidic acid (LPA)/LPA-R/mitogen-activated protein kinase (MAPK) pathway (137, 138). LysoPLs are also substrates for MAGL, whose expression is dysregulated in aggressive PCa (see above). LysoPLs can be reacylated by enzymes such as ysophosphatidylcholine acyl transferases (LPCATs). Grupp and coworkers demonstrated that the expression of lysophosphatidylcholine acyltransferase 1 (LPCAT1), a key enzyme in Lands’ cycle remodeling pathway, correlates with PCa progression and resistance to chemotherapy (i.e., Paclitaxel) and might be used as prognostic biomarker of clinical outcomes and biochemical recurrence (139). LPCAT1 mediates CRPC growth *via* nuclear re-localization and Histone H4 palmitoylation in an androgen-dependent fashion, increasing mRNA synthesis rates. Silencing of LPCAT1 reduced the proliferation and CRPC cell invasive potential, suggesting this enzyme as a potential therapeutic candidate in CRPC (140).

### Alterations in Cholesterol Metabolism

Cholesterol is a major constituent of cell membranes, LDs, and a precursor of androgens synthesis. It is evident that alterations in cholesterol synthesis and metabolism are associated with PCa pathogenesis and progression (141, 142). PCa cells can acquire cholesterol from exogenous sources, including circulating lipoproteins (i.e., VLDL, and LDL) and exosomes, from intra-cellular storage (i.e., LDs), and from *de novo* cholesterol synthesis. All these processes are significantly altered in PCa, especially in aggressive PCa and CRPC. As mentioned above, Yue and coworkers demonstrated an aberrant accumulation of esterified cholesterol in LDs of high-grade PCa and mets due to PTEN loss-mediated activation of the PI3K/AKT pathway, and consequent increase of SREBP and LDL-R (126). However, low levels of LDL-R and high squalene monooxygenase (SQLE) expression were recently detected in high Gleason grade-human PCas and associated with lethal disease. According to these new results, PCas that progress to lethal disease rely on *de novo* cholesterol synthesis (via SQLE), rather than transcellular uptake (via LDL-R) or cholesterol esterification (via Sterol O-Acyltransferase 1, SOAT1) (142). The association of SQLE overexpression with lethal disease was validated in a second study from the same group looking at three different prospective cohorts (143). Absolute SQLE expression was associated with lethal cancer independently of Gleason grade and stage and with increased histologic markers of angiogenesis. SQLE expression at PCa diagnosis was found to be prognostic for lethal PCa both after prostatectomy and in a watchful waiting setting (143). Conversely, vitamin D-regulated catabolic enzyme sterol-27-hydroxylase (CYP27A1), which converts cholesterol to 27-hydroxycholesterol was detected at low levels in tumors characterized by high Gleason grade and high expression of cholesterol synthesis enzymes, including SQLE. Low expression of CYP27A1 was also associated with higher risk of lethal cancer, independent of SQLE (144). Altogether, these data support the notion that intra-tumor cholesterol accumulation (via increased synthesis or reduced catabolism) is a feature of lethal PCa. As expected, the key enzymes for cholesterol synthesis, 3-hydroxy-3-methyl-glutaryl-coenzyme A reductase (HMGCR, the first-rate limiting enzyme) and 3-hydroxy-3-methyl-glutaryl-coenzyme A synthetase (HMGCS) are regulated by androgens and upregulated in PCa, especially in CRPC and contribute to CR [reviewed in (12)]. HMGCS and HMGCR were found overexpressed in stromal cells when co-cultured with PCa cells to support PCa progression, suggesting that HMGCS and HMGCR in both PCa epithelium and stroma, might serve as theraputic targets (145).

## The Role of Lipids as Signaling Mediators in PCa

Besides their function as building blocks and energy suppliers, lipids can function as intra- and extracellular messengers and mediators of malignant behavior. Several classes of lipids are involved in signaling, including sphingolipids and eicosanoids. Tumor-promoting functions have been described for several sphingolipids, including sphingosine, spingosine-1-phosphate (S1P), ceramide, and ceramide-1-phosphate (C1P) (146). By using isotopic FA labeling strategy coupled with metabolomic profiling platforms to comprehensively map palmitic acid incorporation into complex lipids in cancer cells, Louie and coworkers elucidated that cancer cells, including PCa cells, and tumors robustly incorporate and remodel exogenous palmitate into structural and oncogenic glycerophospholipids, but mostly in sphingolipids and ether lipids. FA incorporation into oxidative pathways was reduced in aggressive PCa cells, and instead shunted into pathways for generating signaling lipids such as ceramide and sphingomyelin, suggesting a role for sphingolipids in PCa progression (147). In line with this, Increased levels of S1P were found in more aggressive PC. Pharmacological inhibition (with ABC294640) of sphingosine kinase 2 (SphK2), one of the two Sphk isoforms that catalyzes the synthesis of S1P from sphingosine, effectively reduced CRPC cell proliferation and xenograft tumor growth by targeting AR and the oncogene MYC (148). Classically, ceramide induces senescence and growth inhibition in cancer. However, recent studies suggested that ceramide effects are context dependent and rely on downstream effectors, which can both promote or inhibit tumor growth (149). Along the line, increased expression of acid ceramidase (AC) was observed in PCa. AC significantly altered the expression of ceramide species without affecting the total levels. In AC-overexpressing DU145 cells, low levels of C14-C20 ceramides (long chain ceramides) and elevated levels of C24, C24:1 ceramides (very long chain ceramides) were indeed detected. This was associated with increased proliferation, migration and augmented tumorigenicity *in vivo*, which were reversed by pharmacological or genetic AC inhibition (150, 151). Although AC-mediated oncogenic mechanisms are still unknown, it is likely that AC-induced very long chain ceramide species promote cell growth while long chain ceramides induce cell apoptosis [reviewed in (151)]. Consistently, LC/MS-based lipidomics in plasma from patients with primary PCa, mHSPC, and mCRPC, showed that elevated circulating ceramide levels are associated with poor outcomes across tumor stages progression from localized PCa, mHSPC, to mCRPC. Patients with elevated ceramide levels were more likely to have metastatic relapse, therapeutic failure (ADT/docetaxel), and shorter overall survival. The authors also validated a previously published prognostic 3-lipid signature with potential clinical traslation (152). Both ceramide and C1P are activators of PLA2, an enzyme that releases AA for subsequent conversion to prostaglandins, molecules involved in inflammation, immunity, and tumor growth modulation (see below). Increased levels of prostaglandins, like PGE2, are associated with enhanced PCa proliferation and invasion, which can be reversed by the use of cyclooxygenases (COX) inhibitors, suggesting the involvement of PGE2 in PCa progression. Contrasting results have been however obtained, highlighting the need for more validation studies (153, 154). Phosphoinositides represent another class of critical signaling molecules and central mediators of the PI3K/Akt/mTORC1 signaling axis. Activation of PI3K results in the rapid conversion of PI(4,5)P2 into PI(3,4,5)P3, leading to AKT activation. PI(4,5)P2 it-self can also play a major role in recruiting cytosolic proteins, facilitating processes like fusion, membrane budding, and the formation of signaling platforms [reviewed in (9)]. Finally, glycerolipid-derived mediators, such as DAG, LysoPA and LysoPC are involved in cancer progression. DAG, generated from the hydrolysis of PI(4,5)P2, functions as a second messenger that triggers the oncogenic activation of protein kinase C (PKC). Sustained levels of DAG and activated PKC signaling were reported as a mechanism of resistance to FASN inhibitors, suggesting the assessment of DAG as predictive biomarker of FASN activity and the therapeutic combination of FASN and PKC inhibitors (155).

## Oncogenic and Environmental Regulation of Lipid Rewiring in PCa

Lipid metabolism rewiring is very dynamic. Cancer cells, including PCa cells, adapt their metabolism in response to changes in nutrients supply, hormonal status, growth factors stimuli as well as epi/genetic alterations in oncogenes (i.e., MYC, PI3K/AKT) or tumor suppressor genes (i.e., PTEN, p53, RB), commonly found in mCRPC, as comprehensively described in our recent review (31). Here, we focus on the impact of systemic metabolism and environmental factors, in particular diet, to PCa metabolism rewiring and disease progression.

Both obesity and sustained consumption of fat-enriched diets alter nutrient gradient in the TME, which may favor cancer cells/TME metabolic symbiosis, inflammation, cancer progression, and chemoresistance (10). SFA-enriched diet is sufficient to promote mCRPC in the nonmetastatic PTEN KO mouse model *via* an aberrant lipogenic program orchestrated by SREBP (156). In Hi-MYC mouse model, HFD-induced obesity (enriched for SFA) amplifies a c-MYC-mediated oncogenic transcriptional signature, which is associated with lethality in patients (156–158). Using the same Hi-MYC mouse model, Blando and coworkers also showed that HFD-induced obesity enhances, whereas 30% caloric restriction reduces growth factor (AKT/mTORC1 and STAT3) and inflammatory (NFκB and cytokines) signaling and PCa progression (159). Consistently, reduced dietary fat intake was shown to delay PCa progression to CRPC and to prolong survival in xenograft models, suggesting low-fat diet as a promising adjuvant intervention during ADT (160). Besides SFAs, the ratio between omega-3 (n-3)/omega-6 (n-6) PUFAs also affect PCa progression. Omega-3 but not omega-6 PUFAs slowed down the growth of CRPC in PTEN KO mouse model in part by accelerating proteasome-dependent degradation of AR protein (161). In line with this, an isocaloric 20% kcal fat diet consisting of n-6 and n-3 FAs in a ratio of 1:1 (n-3 diet) reduced tumor growth rates, tumor volumes, and serum PSA levels in LAPC-4 xenografts with respect to n-6 FAs-based diet (n-6 diet). n-3 diet-tumors were characterized by low proliferation, increased apoptosis, and reduced levels of COX-2, PGE-2, and VEGF. Furthermore, LAPC-4 cells proliferation in medium containing n-3 diet serum was reduced by 22% with respect to n-6 diet (162). Several clinical trials are ongoing to evaluate the effect of n-3 PUFA in patients with advanced PCas, as well as in active surveillance and PCa prevention (NCT00458549, NCT03753334, NCT03753334, NCT00253643, NCT02176902, NCT02333435). Results from these studies will be valuable to understand whether nutrition intervention should be implemented in the management of PCa patients prior to or along with ADT/AR signaling inhibitors.

Obesity is also associated with increased fat storage in the adipose tissue. Interestingly, several tumors grow in anatomic proximity to adipose cells. This is the case of PCa, which grows adjacent to the peri-prostatic adipose tissue (PPAT) and develops mets in fatty bone marrow [reviewed in (163)]. Adipocytes can act as driving force to promote PCa cells migration to PPAT. Laurent et al. demonstrated that PPAT-derived adipocytes secrete the chemokine CCL7, which diffuses to the peripheral zone of the prostate, stimulating the migration of CCR3 expressing PCa cells. The latter is reversed by CCR3/CCL7 axis inhibition. In human PCas, CCR3 receptor expression is associated with higher occurrence of aggressive disease with extended local dissemination and biochemical recurrence (164). CCR3 is also potentially involved in the homing of PCa cells to the bone. Using *in vitro* migration assays, the same authors demonstrated that soluble factors released by human primary bone-marrow-derived adipocytes drive the directed migration of PCa cells in a CCR3-dependent manner. Furthermore, Oncomine microarray database uncovered increased levels of CCR3 mRNA in bone mets with respect to primary tumors, while IHC experiments demonstrated overexpression of CCR3 in bone *versus* visceral mets (165). Altogether, this evidence suggests the potential benefit of CCR3 antagonists in the treatment of advanced PCa. In a recent review, Nassar et al. not only describe the role of PPAT as a source of FAs and mitogens but also uncover the existence of a crosstalk between PCa and PPAT that sustains PCa pathogenesis and progression (166). In line with this, MRI-based PPAT measurements have provided new useful information in the prediction of PCa progression. Peri-prostatic fat area (PPFA) and PPFA to prostate area ratio (PPFA/PA) was reported as independent predictor of PCa, lymph node mets, Gleason score, tumor stage, and proliferation index (i.e., Ki-67) (167, 168). Thus, PPFA measurements along with transrectal ultrasound-guided biopsy may improve PCa detection and risk stratification. PPAT volume has been recently also associated with reduced progression-free survival in men with PCa on active surveillance and with poor response to ADT in patients with advanced PCa (169). These results highlight the crucial role of PPAT in PCa progression and the clinical value of MRI-based measurements of PPAT to predict prognosis and therapy response.

## The Role of Lipids in Mediating Tumor-TME Crosstalk

While the role of FAs in promoting inflammation and mediating inflammatory signaling has been largely characterized, more recent data suggest a key role for FAs in immune metabolism [reviewed in (10, 170)]. Both FA synthesis and oxidation are important regulators of immune responses. FA synthesis plays a role in antigen presentation and T cell activation, whereas FAO is a key feature of CD8 memory T cells (170). The source of lipids used for FAO in memory T cells is cell type specific. Central memory CD8 T cells cannot effectively take up lipids and rely on lipolysis for FA supply, whereas tissue resident memory CD8 T cells require uptake of exogenous lipids for their survival and proliferation (171–173). In contrast to CD8 T cells, naïve and memory CD4 T cells require FA uptake and synthesis for full activation and proliferation (174). Regulatory T cells (Treg) and M2-like macrophages rely on lipid-dependent catabolism. Treg cells predominantly use FAO-fueled oxidative phosphorylation (OXPHOS) to generate energy and FAO inhibition with etomoxir suppresses Foxp3 expression in Treg cells without affecting T effector cells (Teff) cells. Thus, Treg cells display a survival advantage in low-glucose and lipid-rich environments over Teff cells and are well adapted to reside in fat tissue and lipid-rich TME, which is consistent with their increased frequency in the TME (175, 176). FAO is also required for the maturation and function of IL-4-induced anti-inflammatory M2 macrophages, which uptake FAs through CD36 and FATP1 to maintain their phenotype (177–180). FAO alterations and LD accumulation are also linked with dendritic cells dysfunction, highlighting the importance of lipids in antigen presentation (181–183).

Michelet and coworkers showed that HFD-induced obesity induces PPAR-driven lipid accumulation in Natural Killer (NK)cells, causing a complete ‘paralysis’ of their cellular metabolism and trafficking, resulting in blunted antitumor responses (184). Similarly, the integration of single-cell RNA sequencing, multiplexed immunofluorescence IHC, and mass-spectrometry approaches *in vivo*, uncovered that HFD-induced obesity impairs CD8^+^ T cell function in TME due to a distinct metabolic adaptation to obesity by the tumor and T cells. While tumor cells increase fat uptake, tumor infiltrating CD8^+^ T cells do not, leading to altered FA partitioning in HFD tumors, which impaired CD8^+^T cell infiltration and function. Analysis of human cancers revealed similar transcriptional changes in CD8^+^ T cell markers, suggesting the potential of lipid metabolism interventions to improve cancer immunotherapy (185). In contrast, obese cancer patients seem to respond to ICI, a phenomenon known as “Obesity paradox” (186). Thus, further studies are needed to clearly understand the impact of obesity and obesogenic HFD on immune therapy efficacy in patients.

Cancer cells not only suppress tumor immune surveillance, but they can also hijack the immune system to support their growth. For instance, ovarian cancer cells promote the efflux of cholesterol from macrophages which in turn drives a pro-tumoral M2 phenotype (187). Moreover, it has been reported that cancer cells can also promote tumor-associated myeloid-derived suppressor cells (MDSCs) to produce PGE2, an oxylipin with immune suppressive functions. This seems to occur through a cancer-dependent increase of Fatty acid transport protein 2 (FATP2) expression, which allow AA transport in MDSCs for PGE2 synthesis (188).

While the role of lipids in PCa immune TME has not been carefully investigated, early preliminary data showed increased expression of immune checkpoint PD-1, PD-L1, and PD-L2 in tumor tissues from PTEN KO mice fed HFD, suggesting an opportunity for ICI (189). Considering that the response to ICI has been so far disappointing, understanding whether obesity may boost response to ICI and “paradoxically” favor the use of immune therapy is crucial to identify a subset of mCRPC patients, who may potentially respond to immune therapies.

## Exploiting Lipid Metabolism Rewiring for Therapeutic Intervention

In light of the aforementioned changes in lipid metabolism during PCa progression, huge efforts have been directed on tackling enzymes and transporters involved in all the aspects of lipid metabolism (from FA uptake transport, *de novo* FA/cholesterol synthesis, sphingolipid and phospholipids synthesis, to lipid storage and lipolysis). Recently published reviews from our group and others have provided an exhaustive description of the small molecules/compounds targeting lipid metabolism tested so far in oncology (9, 31). Here, we emphasize those compounds that have already been approved for clinical use or are currently tested in clinical trials.

### Inhibitors of *De Novo* FA Synthesis

The majority of therapeutic efforts have been focused on FASN, resulting in the development of several FASN inhibitors (i.e., Orlistat, C75, cerulenin, C93, Fasnall) with good results in the preclinical setting. Unfortunately, off-target effects, poor solubility and pharmacokinetics, and untoward side effects, including important weight loss, prevented their clinical translation [reviewed in (31)]. The development of TVB-2640, an orally available inhibitor of FASN β-ketoacyl-reductase domain has changed the perspective. A phase I clinical trial in cancer patients has been completed, showing the safety and efficacy of TVB-2640 in solid malignancies (NCT02223247). Combined with paclitaxel, TVB-2640 provided positive results in heavily pretreated breast cancer patients, while the non-orally available analog TVB-3166 was effective in mCRPC preclinical models (190–192). Phase II trials are now investigating TVB-2640 in several solid tumor types including HER-2 positive advanced breast cancer in combination with trastuzumab. (NCT03032484, NCT03179904, NCT02980029, NCT03808558). Our group also characterized a new oral-available small molecule irreversible FASN inhibitor (IPI-9119) with potential clinical translation. In the preclinical setting, we demonstrated that selective FASN inhibition antagonizes the growth of mCRPC, in part by inducing ER stress-mediated downregulation of AR-FL and AR-V7 protein levels and their transcriptional activity. As a result, IPI-9119 improved the response to enzalutamide in mCRPC cell lines and organoid models. Our data support FASN repression as a non-canonical approach to inhibit AR-V7, thus overcoming current resistance to standard of care for mCRPC. Multiplex immunofluorescence analysis combined with digital pathology of mCRPC tumor microarrays confirmed FASN/AR-V7 co-expression in about 80% of mCRCP patients resistant to enzalutamide and abiraterone, highlighting this patient subset as the ideal candidate for the treatment with FASN inhibitors (22). Carefully designed clinical trials are still needed to adequately define the timing, combinations, and the suitable population to test.

### Inhibitors of FA Oxidation

Targeting FAO in mCRPC has recently gained a lot of attention. Iglesias-Gato and coworkers have recently identified a subgroup of bone mets characterized by elevated expression of FAO enzymes, and thus potentially responsive to FAO inhibitors. These findings also underline the urgent need for adequate patient stratification when metabolic therapies are considered as therapeutic approaches (193). Combinations of FAO inhibitors (etomoxir, ranolazine, and perhexiline) and enzalutamide have been tested in mCRPC cell and xenograft models with positive results. Unfortunately, etomoxir use in the clinical setting has been terminated due to toxic side effects, mostly hepatotoxicity. In contrast, ranolazine and perhexiline are already approved for the treatment of heart diseases in Europe, US, and Australia (194), opening a potential safe avenue for the combinations of FAO and AR signaling inhibitors in mCRPC.

### Inhibitors of Cholesterol Synthesis

Statins are commonly used to lower cholesterol levels and reduce cardiovascular risk. Statins use in the prevention of cancer risk has been evaluated with conflicting results. Their potential use in combination with the standard of care in the treatment of PCa has recently gained attention (31).

A clinical trial designed to test whether atorvastatin (an HMGCR inhibitor) delays the development of CR during ADT in metastatic or recurrent PCas is currently ongoing (NCT04026230). In the preclinical setting, HMGCR inhibition with simvastatin enhances the efficacy of enzalutamide and decreases AR/AR-Vs protein levels *via* inhibition of mTOR pathway (195).

A recent meta-analysis evaluated the effects of statins use on treatment outcomes (i.e., overall survival and cancer‐specific survival) among patients with advanced PCa treated with ADT or AR signaling inhibitors. Statin use was associated with lower risk of all‐cause mortality and cancer‐specific mortality in advanced PCa patients treated with ADT, whereas inconsistent results were obtained with AR signaling inhibitors (196). Thus, future studies are still required to establish the efficacy of statins in combination with AR signaling inhibitors in mCRPC patients.

## Application of Lipidomics and Mass-Spectrometry Imaging in PCa Research

Despite the crucial role of lipid metabolism in PCa progression and resistance to endocrine therapies, lipidomics studies have only recently reached the spotlight most likely due to the methodological challenge of analyzing simultaneously diverse lipid classes and molecular species and technical issues associated with these analytical techniques. An outstanding review has recently highlighted the current advances of lipidomics and mass-spectrometry imaging in cancer research and their critical role in precision medicine (9). Here, we briefly summarize studies using these technologies for the identification of new predictive/prognostic biomarkers in PCa.

Lin et al. performed LC/MS-based lipidomics in plasma samples from a discovery cohort of CRPC patients and identified forty-six lipids, predominantly sphingolipids, associated with poor prognosis. The authors derived a prognostic three-lipid signature (ceramide d18:1/24:1, sphingomyelin d18:2/16:0, phosphatidylcholine 16:0/16:0) as independent prognostic factor (197). More recently, the same group detected elevated circulating ceramide species in association with poorer clinical outcomes across the PCa progression and validated the three-lipid prognostic signature in an independent cohort (152). These studies not only identified an easily detectable prognostic biomarker but also highlighted the crucial role of sphingolipids in PCa progression. Similarly, Butler et al. profiled PCa cell lines, xenografts, and patient-derived explants under treatment with androgen and AR signaling inhibitors. Significant changes in lipid elongation for multiple phospholipid classes in response to androgen treatment were identified and reversed by enzalutamide, suggesting the utility of lipidomics to predict response to endocrine therapies (198). Lipidomics and transcriptomics integration in PCa and adjacent normal tissues also identified a strong accumulation of cholesteryl esters (CEs) most likely due to increased expression of scavenger receptor class B type I (SR-BI). CE accumulation was associated with disease progression and mets formation. In a discovery set, CE robustly differentiated PCa from normal tissue. In a validation set, CEs not only potently distinguished PCa from normal tissue, but it also discriminated PCa from benign prostatic hyperplasia (BPH) superior to PSA, suggesting CE, particularly, cholesteryl oleate, as a biomarker for PCa detection (199). Furthermore, targeted lipidomics in EVs derived from prostate and PCa cell lines uncovered differences in the molecular lipid species associated with PCa progression. These differences highlight the importance of characterizing the EV lipidome, which may lead to improved prognostic biomarkers (200). Despite the high resolution, sensitivity, and specificity of LC/MS-based lipidomics, these technologies fail to provide spatial information and to integrate the information of biomarker expression with tissue pathology and compartment distribution. The development of MSI has overcome this limitation. MSI thus represents an important step forward for the evaluation of metabolic reprogramming occurring in the TME. Matrix-assisted laser desorption ionization (MALDI)-MSI, where the sample is mixed with a UV-absorbing crystalline matrix material and ionized by the laser beam, is the most commonly used MSI method (201). Our group applied MALDI-MSI to investigate changes in lipid metabolism associated with gleason score. We detected increased levels of 31 lipids, including several phosphatidylcholines, PA, phosphatidylserines, phosphatidylinositols, and cardiolipins, in Gleason score 4 + 3 compared with Gleason score 3 + 4, suggesting these analytes as potential biomarkers of PCa aggression worth further validation. Interestingly, we identified lipid changes in both regions of high tumor cell density, and in regions of tissue that appeared histologically “benign”, implying the occurrence of precancerous lipid changes with prognostic significance (202). Using a similar approach, Andersen et al. identified increased levels of metabolites crucial for lipid metabolism in PCa, including metabolites involved in the carnitine shuttle as well as building blocks for *de novo* lipogenesis (203). The feasibility of spatial and rapid detection of metabolites associated with PCa onset and progression showcases MALDI-MSI as a promising and innovative diagnostic/prognosis tool in the clinical setting.

## Discussion

Lipid metabolism rewiring is highly dynamic throughout the course of PCa progression. Intracellular lipid changes due to either environmental cues or *de novo* FA synthesis/FAO increase PCa cells fitness and their capability to adapt to oxidative stress, hypoxia, ER stress, to maintain redox balance, and to counteract ferroptosis and genotoxic insults. Recent evidence also supports the role of lipids as key players in shaping TME metabolism, in particular immune metabolism. This is especially exacerbated by obesity or consumption of HFD diet, conditions in which cancer cells hijack lipids (with the support of tumor-surrounding adipose cells or cancer-associated fibroblasts) for their own benefit, impairing anti-tumor immunity. The rapid advance of lipidomics and MALDI-MSI has allowed to gain, a previously unforeseen, awareness of the dynamicity and adaptability of lipid rewiring during PCa progression, taking into account the influence of systemic metabolism and tumor-TME crosstalk. In the imminent future we anticipate the integration of MALDI-MSI, spatial transcriptomics, and digital pathology will further advance our current understanding of the biology of lipids in PCa progression and will offer opportunities for the identification of new druggable targets. Unfortunately, we still have a long road ahead to validate lipids as biomarkers and to translate the lipid-metabolism targeting drugs available so far in the clinical setting. The journey has started long time ago, but we are now fully equipped with the adequate models (i.e., patient-derived organoids, explants, xenografts, co-culture systems, immune-competent mouse models, etc.), technologies, and bioinformatics support to rapidly move forward.

PCa is “a matter of fats”. The big challenge is to carefully identify and target those lipid and pathways that are tumor-friends while preserving those that protect our health and longevity.

## Author Contributions

All authors contributed to the article and approved the submitted version.

## Conflict of Interest

The authors declare that the research was conducted in the absence of any commercial or financial relationships that could be construed as a potential conflict of interest.

## Publisher’s Note

All claims expressed in this article are solely those of the authors and do not necessarily represent those of their affiliated organizations, or those of the publisher, the editors and the reviewers. Any product that may be evaluated in this article, or claim that may be made by its manufacturer, is not guaranteed or endorsed by the publisher.
